# Retraction: ErHuang formula improves renal fibrosis in diabetic nephropathy rats by inhibiting CXCL6/JAK/STAT3 signaling pathway

**DOI:** 10.3389/fphar.2024.1530375

**Published:** 2024-12-02

**Authors:** Frontiers Editorial Office

The journal retracts the 24 January 2020 article cited above.

Following publication, concerns were raised regarding the integrity of the images in the published figures. Image duplication concerns were identified in Figures 4E and 5C. The authors failed to provide a satisfactory explanation during the investigation, which was conducted in accordance with Frontiers’ policies. As a result, the data and conclusions of the article have been deemed unreliable and the article has been retracted.

This retraction was approved by the Chief Editors of Frontiers in Pharmacology and the Chief Executive Editor of Frontiers. The authors agreed to this retraction.

